# Genetic disorders in beef cattle: a review

**DOI:** 10.1007/s13258-017-0525-8

**Published:** 2017-03-03

**Authors:** Aleksandra Ciepłoch, Karolina Rutkowska, Jolanta Oprządek, Ewa Poławska

**Affiliations:** Department of Animal Improvement, Institute of Genetics and Animal Breeding, Polish Academy of Sciences, Postępu 36A, Jastrzębiec, 05-552 Magdalenka, Poland

**Keywords:** Genetic disorders, Beef cattle, Meat quality, Recessive mutations

## Abstract

The main purpose of present review is to describe and organize autosomal recessive disorders (arachnomelia, syndactylism, osteopetrosis, dwarfism, crooked tail syndrome, muscular hyperplasia, glycogen storage disease, protoporphyria), which occur among beef cattle, and methods that can be applied to detect these defects. Prevalence of adverse alleles in beef breeds happens due to human activity—selections of favorable features, e.g. developed muscle tissue. Unfortunately, carriers of autosomal recessive diseases are often characterized by these attributes. Fast and effective identification of individuals, that may carry faulty genes, can prevent economical losses.

## Introduction

Nowadays identification of animals, which are affected or are carriers of certain disorder is still based on traditional methods, such as pedigree analysis or visual assessment (micro and macroscopic observation). Detection of animals that could spread the faulty gene is crucial, although problematic, because of phenotypic resemblance to normal individuals. The range of phenotypes is wide, due to affected tissues: ophthalmic, nervous, dermal, muscle, skeletal and blood. The defective alleles might be attributed specifically to particular breeds and others to the production type of cattle (meat or dairy). Disorders caused by this alleles are mainly inherited in autosomal recessive manner and are often lethal for homozygous animals. There may be a chance, that one bull carries two distinct affected genes responsible for different disorders. Notwithstanding, breeders propagate individuals without knowledge of their condition, due to their profitable features (developed muscularity, high milk production, increased milk protein and fat content). Unfortunately, these animals often appear to be carriers of genetic defect. This human interference leads to artificial selection resulting in high mortality in herds, reduced reproduction and altered efficiency and quality of meat production. This situation generates economical losses that may be difficult to compensate. The skeletal disorders, such as syndactylism, arachnomelia, osteopetrosis or osteopetrosis with gingival hamartomas generate many losses among beef cattle due to poor muscle development, necessity of culling affected individuals because of their maladjustment to life and even risk of death of dams during birth of the affected calf (Drogemuller et al. 2009; Stark and Savarirayan 2009). Different forms of dwarfism lead to abortion or death of animals not long after birth and often they are associated with lowered immune system response (Sartelet et al. 2012a; Whitlock et al. 2008). Muscle tissue disorders - muscular hyperplasia, crooked tail syndrome are desirable for breeders due to increased muscle mass, but unfortunately they are connected with e.g. low reproduction score, weak skeletal system, organs failure or nervous system disorders, which might result in reduction of animals number in herd (Bouyer et al. 2014; Handschin et al. 2007; Sartelet et al. 2012b). The glycogen storage diseases are metabolic disorders among which type II and V are the most popular in beef cattle. Individuals with these defects show muscle pain and weakness, as well as, problems with motoric condition. Same cases might be even lethal (Citek et al. 2007; O’Rourke et al. 2006; Zlotowski et al. 2006). Another inherited beef trait is connected with blood and it is called the protoporphyria. This disease is very painful for the animal and in the final step leads to the liver damage and, therefore, losses for the breeder (Jenkins et al. 1998). The examples of mutation of genes responsible for chosen genetic disorders are presented in Table 1. Disorders have been chosen according to Online Mendelian Inheritance in Animals catalogue (OMIA) records by such categories as known key mutation, commonly known and used beef breeds, number of publications referring to autosomal recessive disorders inherited among beef cattle, number of papers published recently (since 2000) (Nicholas 2003).

**Table 1:** Mutations in genes responsible for inherited disorders in beef cattle

Disorder	Gene	Mutation	Chromosome	References
Syndactylism	*LPR4* (*MEGF7*)	c.241G>A c.3595G>A c.5385+1G>A	15	Drogemuller et al. (2007) Johnson et al. (2006)
Osteopetrosis	*SLC4A2*	~2,8 kb deletion	4	Meyers et al. (2010)
Osteopetrosis with gingival hamartomas	*CLCN7*	c.2244G>C c.2248T>C c.2250C>A	25	Sartelet et al. (2014)
	*OSTM1*	–	25	Sartelet et al. (2014)
Arachnomelia	*MOCS1*	c.1224_1225delCA	23	(Buitkamp et al. 2011)
Proportionate dwarfism with inflammatory lesions	*RFN11*	c.124_2A>G	3	Sartelet et al. (2012a)
Aggrecan type dwarfism	*ACAN*	c.2266_2267insGGCA c.-198C>T	21	Cavanagh et al. (2007)
Chondrodysplasia	*EVC2 (LBN)*	c.2993_2994ACdel	6	Murgiano et al. (2014) Muscatello et al. (2015)
Dwarfism in Angus cattle	*PRKG2*	c.2032C>T	6	Koltes et al. (2009)
Crooked tail syndrome	*MRC2*	c.2904_2905delAG c.1906T>C	19	Fasquelle et al. (2009) Sartelet et al. (2012b)
Muscular hyperthrophy	*MSTN*	c.821del11 c.938G>A c.871G>T c.3811T>G c.282C>A	2	O’Rourke et al. (2013) Short et al. (2002) Marchitelli et al. (2003) Bouyer et al. (2014) Esmailizadeh et al. (2008)
Glycogen storage disease type II	*GAA*	c.2454_2455delCA c.1057_1058delTA c.1783C>T c.1351C>T c.2223G>A	19	Dennis et al. (2000) Citek et al. (2007)
Glycogen storage disease type V	*PYGM*	c.1468C>T	29	Citek et al. (2007)
Protoporphyria	*FECH*	c.1250G>T c. 1252C>A c.1258C>T	24	Jenkins et al. (1998)

## Syndactylism

The bovine syndactylism is heritable disorder also known as mulefoot disease (MFD) (OMIA 000963-9913). This malformation has an autosomal recessive character, and it occurs differently in each case. This is due to incomplete penetrance and variable expression of this trait (Johnson et al. 2006). This genetic disorder is a non-division or fusion of digits, and it mostly appears as synostosis of phalanges. However, more proximal limbs might also be altered. Mulefoot disease might affect only one foot, as well as all 4 feet. Though, there are observed two gradients: front-rear and right-left. More specifically, right front limb is affected predominantly, then left front, right rear and left rear limb. Moreover, this defect is associated with hyperthermia resulting from increased environment temperature (Yadegari et al. 2013). Syndactylism can be present in both dairy and beef cattle. There have been noted cases of this defect in Holstein, Brown Swiss, Simmental, Hariana, Angus, Hereford, Chanina, Japanese Native, Swedish Red Pied, Czech Black Pied, German Fleckvieh, Danish cattle (Agerholm et al. 1993; Charlier et al. 1996; Leipold et al. 1998).

During studies under mutation causing mulefoot disease there have been many candidates (Duchesne et al. 2006), but eventually one gene has become a strong one—*LRP4* (low density lipoprotein receptor-related protein 4 gene) (Drogemuller et al. 2007; Duchesne et al. 2006). Protein encoded by this gene plays important role in digits development in mammals (Simon-Chazottes et al. 2006). *LRP4* is also known as *MEGF7*—multiple epidermal growth factor-like domains 7 gene (Drogemuller et al. 2007). This gene has been mapped to telomeric end of chromosome 15 using identity-by-descent mapping (IBD) (Charlier et al. 1996).

The molecular basis of syndactylism differ between dairy and beef cattle, although they occur in the LRP4 and impair the formation of LRP4 protein. In beef cattle detected mutations have been specific to breed. In Simmental cattle affected with syndactylism two point mutations are present—first one occurs in exon 3 as substitution of guanidine to adenine (c.241G>A) at the position 42 and it leads to replacement of glycine to serine at the 81 residue, and second one appears at position 59 of exon 26 as substitution of guanidine to adenine (c.3595G>A) resulting in exchange of glycine to serine at the position 1199 (Drogemuller et al. 2007). In Angus cattle point mutation occurs at the first nucleotide of the intron 37 and it is a substitution of guanine to adenine (c.5385+1G>A) (Johnson et al. 2006).

As mentioned before, mutations in gene *LPR4* lead to bone malformations. This may alter the quality of the meat—impaired bone development can cause the loss of weight of the caracass. Additionally, protein LPR4 participates in cholesterol metabolism and there has been formed hypothesis that mutation in *LPR4* exerts changes on yield grade of the beef meat (Karisa et al. 2013).

On account of still not discovered all mutations responsible for bovine syndactylism there has been no molecular diagnostic test developed (Whitlock et al. 2008). In some cases syndactylism can be detected in 37–40th day *post coitum* using autoradiography method (Duchesne et al. 2006).

## Types of osteopetrosis

This group of diseases, also known as ‘marble bone’, are a rare recessively inherited disorders and it are characterized by osteoclast failure and defective bone resorption especially of primary spongiosa. The main characteristic of these disorders are increased skeletal mass and probability of bone fractures, as well as, neurological complications caused by compressing of cranial nerves (Stark and Savarirayan 2009). To distinguish them from other sclerosing diseases of bone, the term *osteopetrosis* is designed only to the group of diseases in which osteoclast defects are apparently demonstrable (Tolar et al. 2004).

There are many clinical characteristics of this group of disorders. The activity of osteoclast is defective, cells that resorb bone are multinucleated and resulting in accumulation of primary spongiosa in marrow cavities, leading to formation of extremely dense and fragile bones (Segovia-Silvestre et al. 2009). Affected calves are almost always stillborn, slightly premature, have flat skull, shortened mandible, impacted molars, protruded tongue and small body size (Teitelbaum 2000).

Historically, the carrier bull was identified in 1964. There were many cases of this defect among the Aberdeen and Angus breed in North America between 1960s and the early 1980s (O’Toole et al. 2012). Two types of this disease occur among breeds of beef cattle—osteopetrosis and osteopetrosis with gingival hamartomas, and they appear in Angus, Red Angus (OMIA 000755-9913) and Belgian Blue (OMIA 001887-9913) cattle, respectively (Meyers et al. 2010; Sartelet et al. 2014). Nevertheless, it was also reported in Simmental, Hereford and Dutch Holstein-Friesian breed (Nietfield 2007).

### Osteopetrosis

In the Red Angus cattle it is a result of *SLC4A2* gene mutation on the bovine chromosome 4 (BTA4) (Meyers et al. 2010). This gene encodes the anion exchanger protein located on the osteoclats membrane, which is responsible for a removal of excess carbonate ions and supply of chloride ions at the time of lacunar acidification (Josephsen et al. 2009). The mutation includes deletion of 2781 bp resulting in removal of one-third of intron 1, the entire sequence of exon 2 and intron 2, and nearly half of exon 3, respectively. Lack of SLC4A2 protein expression prevents bone resorption and resulting in lack of acidification. It can be identify in beef cattle by simple PCR amplification of DNA fragment carrying the causative mutation (~2,8 kb) in *SLC4A2* (Meyers et al. 2010).

### Osteopetrosis with gingival hamartomas

In Belgian Blue cattle the disorder is caused by mutation in other two genes *CLCN7* and *OSTM1* localized on the bovine chromosome 25 (BTA25). These genes are significant due to encoding the chloride-proton (Cl^−^/H^+^) exchanger CIC-7 and its β-subunit Ostm-1 being an osteopetrosis-associated transmembrane protein 1 (Leisle et al. 2011). Of note, there have been identified by NGS three substitutions in exon 23 of the *CLCN7* gene—one of them is silent (c.2244G>C) and two are missense (c.2248T>C, c.2250C>A). Intriguingly, the mutation alters expression level of CIC-7 and accelerate its gating kinetics. To find mutations mentioned earlier there can be performed the Sanger sequencing (Sartelet et al. 2014).

## Arachnomelia

Another skeletal congenital disorder is the arachnomelia, also known as spider legs. Arachnomelia is autosomal recessive mutation defect with complete penetrance (Buitkamp et al. 2008; Drogemuller et al. 2010). Affected individuals reveal legs deformation (prolonged and fragile limbs bones), spinal column malformations (kyphosis and scoliosis) and skull malformations (short lower jaw, rotation in the anterior cranium, ‘pointer head’) (Buitkamp et al. 2008). This malformation is lethal for homozygous animals - calves die around birth. Moreover, dam which gives birth to the deformed calf is at the high risk due to the severe damages (Drogemuller et al. 2009). Carriers of this trait show no clinical symptoms (Drogemuller et al. 2010). This disorder has been found mainly in Brown (i. a. Swiss Brown, Italian Brown), Simmental (i.e. Austrian Simmental, German Simmental) and occasionally in Holstein cattle (Buitkamp et al. 2008; Seichter et al. 2011; Testoni and Gentile 2004).

There have been conducted studies to evaluate which gene is responsible for bovine arachnomelia. It has occured that different genes are involved in this malformation in Brown cattle (OMIA 000059-9913) and Simmental cattle (OMIA 001541-9913). Arachnomelia in Simmental cattle has been mapped to bovine chromosom 23 using linkage analyses by Buitkamp et al. (2008). Mutation which causes ‘spider legs’ is two bp deletion in gene *MOCS1* in the exon 11 causing a frame-shift and premature stop codon. The product of transaltion of this gene is MOCS1 protein which has two domains—elongator protein 3 (Elps) and molybdopterin cofactor C (MoaC). MoaC plays important role in molybdenum cofactor (Moco) biosynthesis, which is a major element of oxidoreductases that participate in carbon, sulphur and nitrogen transformations. It is suggested that due to arachnomelia mutation (c. 1224_1225delCA) the domain MoaC loses its function to synthesize Moco (Buitkamp et al. 2011; Jiao et al. 2013). Moreover, this links directly with defect described in Brown cattle, due to Moco, which is indispensable in sulfite oxidase—mutation in gene *SUOX*, that leads to loss of function of this enzyme, results in arachnomelia within this cattle breed (Drogemuller et al. 2010). Jio et al. (2013) developed PCR-RFLP test with the usage of *Dra*III which detects mutation in gene *MOCS1*.

## Types of dwarfism

This group is one of the most popular defects among beef cattle. There are a few types of dwarfism but all of them can be divided into two groups—proportionate and disproportionate. First one, also called pituitary or ateliotic dwarfism, is distinguished by small but proportionate stature. In most cases this kind of malformation is associated with hypothalmic-growth hormone-insulin-like growth factor axis. Formation of bones and soft tissues are reduced which results in infantile phenotype. Second one include chondrodysplasia, chondrodystrophy and achondroplasia which may be manifested by disproportion in limbs (i.e. rhizomelia), changes in cranial development (flat and broad head), altered endochondral ossification, excessive amount of soft tissue due to its unaffected growing (Latter et al. 2006; Murgiano et al. 2014).

For different types of dwarfism are responsible different genes and mutations. However, opinions are divided whether it is a disorder inherited in autosomal recessive manner or autosomal incomplete dominant manner. This is due to phenomenon that heterozygous animals show symptoms of this defect. Besides carriers of this malformation display features that are characteristic for some breeds and desirable for breeders (high meat yield). In most cases homozygous affected individuals are aborted or die right after birth, not infrequently because of decreased immunity (Whitlock et al. 2008).

### Proportionate dwarfism with inflammatory lesions

One of gene in which occurs mutation responsible for proportionate dwarfism is *RFN11* placed on chromosome 3. Causative mutation appeared to be transition of A to G (c.124_2A>G) and it alters the intron 1 acceptor site of mentioned gene. *RFN11* encodes RING finger protein 11—a subunit of A20 ubiquitin-editing complex which regulates NF-κβ signaling. Due to mutation altered protein loses its functions and individuals are prone to infections and show retarded growth. This defect is specific for Belgian Blue (OMIA 0001686-9913) cattle and its founder is considered to be sire Galopeur des Hayons. For detection of this mutation there may be used quantitative RT-PCR, 5′-exonuclease assay, capillary sequencing (Sartelet et al. 2012a).

### Aggrecan type dwarfism

Other kind of this malformation is bulldog dwarfism which occurs among Dexter cattle (OMIA 001271-9913). This is one of the most famous disproportionate forms of dwarfism and it is caused by mutations in *ACAN* on bovine chromosome 21. These mutations are 4-bp insertion in exon 11 (c.2266_2267insGGCA) and transition of C to T in exon 1 (c.-198C>T) and are called BD1 and BD2, respectively. *ACAN* encodes aggrecan—an aggregating proteoglycan which is an important structural component of cartilage. Mutations BD1 and BD2 cause significant reduction of the product or even its absence due to complete change in its structure (product does not resemble agrrecan), hence cartilage growth is retarded which results in impairment of long bones development. To detect these mutations there can be applied PCR, PCR-RFLP with *Hsp*92I for BD1 and BD2, respectively. Also radiography technique can show differences between normal, affected and carrier animal (Cavanagh et al. 2007).

### Chondrodysplasia

Another disproportionate dwarfism, chondrodysplasia (OMIA 000187-9913), is caused by mutation in *EVC2* gene on chromosome 6. Causative mutation is 2-bp deletion (c.2993_2994ACdel) was discovered in exon 19 of this gene in Tyrolean Grey cattle. *EVC2*, also named *LBN*, codes protein Ellis-van Creveld syndrome 2 protein that transmembrane compound of the basal body of the primary cilia. Mutation leads to frameshift and premature codon stop and it is still unknown either the product is not expressed at all or it just loses its functions due to structural alterations. However, this variation results in impaired cranium, vertebra and limbs development. Application of radiography technique, SNP array genotyping and Sanger sequencing allows to find this deletion (Murgiano et al. 2014; Muscatello et al. 2015).

### Dwarfism in Angus cattle

There has been discovered nonsense mutation causing disproportionate dwarfism in Angus cattle (OMIA 001485-9913) and it appears in *PRKG2* on chromosome 6. Product of *PRKG2* expression is cGMP-dependant type II protein kinase. Although its functions are not well studied it is known to be essential signal transduction pathway in many tissues, such as growth plate cartilage. Causative mutation is a transition of C to T (c.2032C>T) localized in exon 15 and it leads to premature codon stop in kinase domain. Furthermore, this variation changes expression levels of *COL2* and *COL10* that play a key role in growth plate development(Koltes et al. 2009). There is no method of detection described in literature, though, knowing causative mutation sequencing methods can be applied. However, there has been described other type of dwarfism in Angus cattle. It is a proportionate dwarfism but its molecular basis are still unknown (Latter et al. 2006).

## Crooked tail syndrome

Crooked tail syndrome (CTS) (OMIA 001452-9913) is a disorder connected with muscular and skeletal deformations. Its symptoms are increased muscle mass, retarded stature, thickset head, scoliosis and short fore limbs, which are straight. Concomitantly, spastic paresis appears. This syndrome is observed among Belgian Blue cattle. The carriers of this mutation, which has been estimated to approximately 25% of Belgian Blue cattle, are desirable because of their muscular development, but, unfortunately, this has induced a spread of this syndrome among this breed population (Fasquelle et al. 2009; Sartelet et al. 2012b).

The cause of CTS are mutations in mannose receptor C type 2 (*MRC2*) gene, which has been mapped to bovine chromosome 19. *MRC2* encodes one of the mannose receptors—180 kDa endocytic transmembrane glycoprotein (Endo108) (East et al. 2003). Endo 180 participates in bones development and regulation of remodeling and degradation of extracellular matrix—interacts with plasminogen activation system, has ability to bind collagen and activity of C-type lectin. It has very complex structure—contains i.a. eight C-type lectin-like domains (CTLDs), carboxyterminal cytoplasmic domain and stop-transfer signal. One of the mutations is a double deletion of adenine and guanine (c.2904_2905delAG) in exon 20 of *MRC2*. It leads to frame-shift, which results in loss of three CTLDs domains (CTLD6, CTLD7 and CTLD8), C-terminal cytoplasmic domain and stop-transfer signal in Endo 180. Due to these alterations, glycoprotein loses its endocytic functions and cannot incorporate into the plasma membrane (Fasquelle et al. 2009). The second mutation occurs in exon 13 and it is a substitution of thymine to cytosine (c.1906T>C) and it is suggested that it leads to exchange of cysteine to arginine in the codon 636, which results in alteration in CTLD3, which is indispensable for stabilization of Endo 180 structure (Sartelet et al. 2012b).

Fasquelle et al. (2009) have developed 5′exonuclease assay to detect mutation c.2904_2905delAG. They have also used Western blotting to assay Endo180. There has been used polyclonal rabbit antybody CAT2, which has shown sufficient specificity and enabled to differentiate between mutants and non-affected individuals. This test has also given results pointing that carriers have half of level of this glycoprotein as compared to the normal animals. Sartelet et al. (2012a, b) have also evolved 5′exonuclease test for detection of c.1906T>C mutation.

## Muscular hyperplasia

Muscular hyperplasia, also known as double muscling (DM) (OMIA 000683-9913), is an inherited condition that results from an increase in a number of muscle fibers. This condition is also, incorrectly, called hypertrophy (Bouyer et al. 2014). It was first reported by Culley in 1807 and then by Youatt in 1834 (Short et al. 2002). The occurance of disease is observed in many beef cattle breeds including Belgian Blue, Piedmontese (Gengler et al. 1995), French Blonde d`Aquitaine, Asturiana de los Valles (Olivan et al. 2004), Nellore (Grisolia et al. 2009), Limousin (Esmailizadeh et al. 2008; Sellick et al. 2007) and Marchigiana (Marchitelli et al. 2003).

Animals with DM phenotype present many clinical syndromes. They are characterized by extreme high carcass yield, what is connected with reduction in the size of most vital organs such as heart, lungs, kidneys, as well as, high frequency of broken bones. Whereby, they are more susceptible to respiratory diseases, urolithiasis, alveolar hypoxia, hypoxemia and dystocia in comparison to normal cattle. Moreover, endurance of double muscled cattle is less than in normal animals, leading quickly to exhaustion after severe exercise, because it is associated with reduction of mitochondrial gene expression (Handschin et al. 2007). In addition, digestive tract and feed intake capacity is decreased (Olivan et al. 2004). Quick muscle growth has negative impact on reproduction of cattle including reduction of female fertility, abortion and stillbirth of calves increased stress susceptibility and it was reported that embryos have a higher mortality rate (Bouyer et al. 2014).

Myostatin (MSTN), also known as GDF8 (growth and differentiation factor 8), is included to the superfamily of transforming growth factor *β* (TGF-*β*). It functions as a negative regulator of skeletal muscle mass development by inhibiting the Myo5 and MyoD factors, that play a pivotal role in muscle development and maturation (McPherron et al. 1997). Generally, myostatin gene is located at the centromeric end of the bovine chromosome 2 (BTA2) (Charlier et al. 1995; Grobet et al. 1997; Smith et al. 1997) and remained highly conserved throughout evolution, consisting of three exons and two introns whereas the third exon encodes entire bioactive COOH– terminal domain (Lee and McPherron 2001). Moreover, myostatin is synthesized as a form of precursor, which subsequently dimerizes via disulfide bonds. Interestingly, activation of myostatin occurs by proteolytic cleavage by some kind of the BMP 1 tolloid family of metalloproteinases, respectively (Bouyer et al. 2014).

Approximately 20 different types of polymorphisms of myostatin gene—*MSTN* such as deletions, substitutions or insertions have been identified in European cattle breeds what indicates high rate of mutation, mostly in non conserved regions. However, nowadays six different mutations, which are related to double muscling syndrome, are known (Grisolia et al. 2009). The best characterized mutation was recognized in Belgian Blue cattle and consists of 11 bp deletion in the third exon of nucleotides 821–831 inclusive (c.821del11) (O’Rourke et al. 2013), leading to a frame shift and subsequently inducing premature termination of translation. Moreover, the same mutation is responsible for double muscling in Asturiana cattle (Bellinge et al. 2005). On the contrary, in Piedmontese cattle transition of G>A altering cysteine to tyrosine at position 938 in the myostatin gene (c.938G>A) (Short et al. 2002) is present. Interestingly, other reported mutation occurring in Marchigiana breeds includes transversion of G>T nucleotide at position 871 (c.871G>T), what determines the stop codon (Marchitelli et al. 2003). In Blonde d’Aquitaine, point mutation occurs in position 3811 (c.3811T>G) (Bouyer et al. 2014). Of note, Limousin breeds present C>A substitution at position 282 resulting in alteration of leucine to phenyloalanine (c.282C>A). However, describing variant has a less severe effect on the muscling phenotype in comparison to other mutations (Esmailizadeh et al. 2008).

To detect the myostatin polymorphism quantitative RT-PCR, Sanger sequencing (Bouyer et al. 2014), PCR with 3.5% agarose gel (Gill et al. 2009), PCR-RFLP (Sellick et al. 2007) and Northern analysis are used (Kambadur et al. 1997).

Despite all defects connected with this mutations, the DM cattle have many positive traits as regards to carcass, because they have reduced content of total fat, higher PUFA:SFA ratio and higher conversion of omega-3 fatty acids in compared to normal animals (Sevane et al. 2014). Meat from affected individuals has also a lower content of collagen and connective tissue, what implies more tender and leaner meat (Sanudo et al. 2004). Due to the low concentration of myoglobin, meat of DM cattle has lighter colour. Moreover, it has high moisture and protein content (Olivan et al. 2004).

From farmers and consumers point of view double muscled animals are very important in livestock husbandry, whereby frequency of DM animals has increased especially in Europe countries (Olivan et al. 2004). This situation is due to higher consumers preferences according to meat quality, especially less content of fat (Biagini and Lazzaroni 2005).

## Types of glycogen storage disease

There are many types of glycogen storage disease, but among cattle type II and V are the most common. Both of them are inherited as recessive autosomal disorder and occur among beef cattle (Citek et al. 2007; Zlotowski et al. 2006).

### Glycogen storage disease type II

Glycogen storage disease type II (GSD II) (OMIA 000419-9913) is also known as generalized glycogenosis, Pompe’s disease or acid maltase deficiency (Citek et al. 2007; Moreland et al. 2012). It is the only one among glycogen storage disease which has a lysosomal character. Pompe’s disease appears due to deficiency of acidic α-glucosidase (GAA) activity, that results in glycogen accumulation in lysosomes. GAA (EC 3.2.1.20) participates in conversion of glycogen, maltose and isomaltose to glucose and it catalyzes hydrolysis of α-1,4 and α-1,6 bonds (Dennis et al. 2000). The deficiency of this enzyme might be displayed in lymphocytes, muscles and liver. Affected individuals, show progressive muscle weakness, problems with motoric coordination and growth. Microscopic observations reveals severe vacuolation especially in skeletal muscles and myocardium. In some cases these symptoms may not be exposed right after birth—the period of disclosing these features is up to 6 months, however homozygous animals live less than 12 months (Zlotowski et al. 2006). This disease affects Brahman and Shorthorn cattle (Citek et al. 2007; Zlotowski et al. 2006).

GSD II occurs due to mutations in gene *GAA* encoding acidic α-glucosidase, which is located on bovine chromosome 19, although different mutations have been found among breeds. In Shorthorn cattle, the cause of this disorder is dinucleotide deletion in exon 18 (c.2454_2455delCA), which is lethal. In Brahman cattle, the main causes of GSDII are dinucleotide deletion in exon 7 (c.1057_1058delTA), resulting in frameshift, and transition of cytosine to thymine in exon 13 (c.1783C>T). Both of these mutations lead to stop codons. However, there are other mutations attributed to this disease—transition of cytosine to thymine in exon 9 (c.1351C>T) and transition of guanine to adenine in exon 16 (c.2223G>A). The first one results in decreased level of acidic α-glucosidase activity, and the second one is silent mutation (Citek et al. 2007; Dennis et al. 2000).

There have been developed PCR-RFLP tests to detect heterozygous and homozygous animals for GSDII. In Shorthorns, there has been used *Drd*I restriction enzyme (Dennis et al. 2000). To detect mutations in Brahmans, there have been applied *Bgl*I and *Bsi*EI for mutations in exons 7 and 13, respectively. *Bgl*I has also been used in detection of mutation in exon 9 (Citek et al. 2007; Dennis et al. 2000). *Msp*I has been useful in finding silent mutation in exon 16 (Dennis et al. 2000). Nevertheless, other, non-genetic tests are known e.g. blood mononuclear cell test (enzyme-based test), light and electron microscopy, lectin-histochemistry assay (Zlotowski et al. 2006).

### Glycogen storage disease type V

Glycogen storage disease type V (GSD V) (OMIA 001139-9913), also known as myophosphorylase deficiency or deficiency of muscle glycogen phosphorylase, is a disorder attributated to muscles and it has autosomal recessive character (Citek et al. 2007). Glycogen phosphorylase (EC 2.4.1.1) is responsible for releasing glucose from glycogen by α-1,4 bond cleavage. During that reaction glucose-1-phosphate is produced, which is indispensable in glycolysis (Greenberg et al. 2006). Animals with deficiency of this enzyme show symptoms of muscle pain and, therefore, they are not able to withstand exercises. Moreover, after physical effort their urine is brown and transparent and the level of plasma creatine kinase is increased (Soethout et al. 2002). According to previous studies, this disorder affects solely Charolais cattle and its consequences may be lethal (Citek et al. 2007; O’Rourke et al. 2006).

The cause of GSD V is point mutation in gene *PYGM*, which encodes muscle glycogen phosphorylase and is located on bovine chromosome 29. Transition of cytosine to thymine in position 1468 (c. 1468C>T) in exon 12 leads to exchange of tryptophan for arginine in codon 490 (UniProtKB/Swiss-Prot: P79334.3), which results in production of dysfunctional polypeptide (O’Rourke et al. 2006). This mutation might be detected by usage of PCR-RFLP test with application of *Sty*I (Citek et al. 2007; O’Rourke et al. 2006).

## Protoporphyria

The bovine protoporphyria or bovine ferrochelatase (OMIA 000836-9913) deficiency is inherited as recessive trait. It is characterized by decrease in activity of enzyme taking part in heme biosynthesis—ferrochelatase (FECH; EC 4.99.1.1). FECH has been found on the inner mitochondrial membrane and catalyzes linkage of Fe(II) and protoporphyrin IX, which is the final step of heme formation. Therefore, while protophorphyria accumulation of protophorphyrin occurs and it might be manifested by increased level of prothophorfirin in blood and feces. Due to photoreactivity of this compound, subsequently, photosensitivity appears in individuals affected by this defect, which causes i.a. ulceration, alopecia (mostly nostrils and earlobes lesions), pain. Next to this symptom there can arise liver damage and reduction in productivity (Armstrong et al. 2002; Jenkins et al. 1998; Shibuya et al. 1995). Within carriers, FECH activity is lower than in normal animals, although it is adequate to ward off high concentration of protoporphyrin (Jenkins et al. 1998). This condition may be found among Charolais, Limousin, Blonde d’Aqutaine cattle (O’Rourke et al. 2006; Shibuya et al. 1995).

Mutation, which causes bovine protoporphyria occurs in gene *FECH* that encodes ferrochelatase, that has been mapped to chromosome 24 (NCBI Gene ID: 281158). It is transversion of guanine to thymine appearing in exon 11 at 1250 position that leads to conversion of stop codon to leucine (TGA→TTA; p.X417L). Because of this alteration the final product of translation is 27 amino acids longer. Additionally, downstream from the original stop codon there appear three substitutions—cytosine to adenine at position 1252, guanine to adenine at position 1257 and cytosine to thymine at position 1258. These three additional point mutations are not responsible for causing protoporphyria, because they are not in the coding region. Nevertheless, substitutions c. 1252C>A and c.1258C>T determine significant change in the protein, that has been already modified by the mutation c.1250G>T and due to that, there are some suggestions, that one or both of these substitutions have an impact on the decrease in FECH activity (Jenkins et al. 1998).

There have been developed some tests to detect bovine protoporphyria. To determine whether animal is affected, carrier or normal individual, there can be used PCR-RFLP assay with the usage of *Hpa*I (O’Rourke et al. 2006) or ASPCR (Jenkins et al. 1998). There can also be applied a fluorimetric method for measure ferrochelatase activity by quantifying disappearance of porphyrin (Shi and Ferreira 2003), k-ELISA (Straka et al. 1991) and Western blot analysis (Jenkins et al. 1998; Straka et al. 1991).

## Conclusions

It is commonly known that control of breed condition reduces economical losses. Genetic disorders can cause mortality and decrease in reproduction. In this field there are still applied traditional methods, such as pedigree analysis, visual assessment (micro and macroscopic observation) or even culling. In this work authors attempted to set together molecular methods, which bring new and more accurate approach of identification of genetic defects. The most popular detecting method, for almost all described in this review disorders, are PCR-based tests, due to its accuracy, simplicity and short time of analysis (Table 2). Although, these assays cannot be used in all known genetic disorders, because not all of them are well understood. However, authors goal was to collect and present all data in one paper and clarify, which gene, type and position of mutation are responsible for certain genetic trait.

**Table 2:** Analytical methods of identification of mutations in genes responsible for inherited disorders in beef cattle

Disorder	Analytical method	References
Syndactylism	Autoradiography method (in some cases)	Duchesne et al. (2006)
Osteopetrosis	PCR	Meyers et al. (2010)
Osteopetrosis with gingival hamartomas	Sanger sequencing	Sartelet et al. (2014)
Arachnomelia	PCR-RFLP with application of *Dra*III	Jiao et al. (2013)
Proportionate dwarfism with inflammatory lesions	qPCR 5′-Exonuclease assay capillary sequencing	Sartelet et al. (2012a)
Aggrecan type dwarfism	PCR PCR-RFLP Radiography technique	Cavanagh et al. (2007)
Chondrodysplasia	Radiography technique SNP array genotyping Sanger sequencing	Murgiano et al. (2014), Muscatello et al. (2015)
Dwarfism in Angus cattle	–	–
Crooked tail syndrome	5′exonuclease assay for mutations detection Western blot analysis	Sartelet et al. (2012b), Fasquelle et al. (2009)
Muscular hyperthrophy	qPCR Sanger sequencing PCR PCR-RFLP Northern analysis	Bouyer et al. (2014), Gill et al. (2009), Sellick et al. (2007), Kambadur et al. (1997)
Glycogen storage disease type II	PCR-RFLP with application of *Drd*I, *Bgl*I, *Bsi*EI, *Msp*I	Dennis et al. (2000)
Glycogen storage disease type V	PCR-RFLP with application of *Sty*I	O’Rourke et al. (2006), Citek et al. (2007)
Protoporphyria	PCR-RFLP with application of *Hpa*I ASPCR Fluorimetric method k-ELISA Western blot analysis	O’Rourke et al. (2006), Jenkins et al. (1998), Shi and Ferreira (2003), Straka et al. (1991)

## References

[CR1] Agerholm JS, Basse A, Christensen K (1993). Investigations on the occurrence of hereditary diseases in the Danish cattle population 1989–1991. Acta Vet Scand.

[CR2] Armstrong S, Jonsson N, Barrett D (2002). Bovine congenital erythrocytic protoporphyria in a Limousin calf bred in the UK. Vet Rec.

[CR3] Bellinge RHS, Liberles DA, Iaschi SPA, O’Brien PA, Tay GK (2005). Myostatin and its implications on animal breeding: a review. Anim Genet.

[CR4] Biagini D, Lazzaroni C (2005). Carcass dissection and commercial meat yield in Piemontese and Belgian Blue double-muscled young bulls. Livest Prod Sci.

[CR5] Bouyer C, Forestier L, Renand G, Oulmouden A (2014). Deep intronic mutation and pseudo exon activation as a novel muscular hypertrophy modifier in cattle. PLoS One.

[CR6] Buitkamp J, Luntz B, Emmerling R, Reichenbach HD, Weppert M, Schade B, Meier N, Gotz KU (2008). Syndrome of arachnomelia in Simmental cattle. BMC Vet Res.

[CR7] Buitkamp J, Semmer J, Gotz KU (2011). Arachnomelia syndrome in Simmental cattle is caused by a homozygous 2-bp deletion in the molybdenum cofactor synthesis step 1 gene (MOCS1). BMC Genet.

[CR8] Cavanagh JA, Tammen I, Windsor PA, Bateman JF, Savarirayan R, Nicholas FW, Raadsma HW (2007). Bulldog dwarfism in Dexter cattle is caused by mutations in ACAN. Mamm Genome.

[CR9] Charlier C, Coppieters W, Farnir F, Grobet L, Leroy PL, Michaux C, Mni M, Schwers A, Vanmanshoven P, Hanset R (1995). The mh gene causing double-muscling in cattle maps to bovine chromosome-2. Mamm Genome.

[CR10] Charlier C, Farnir F, Berzi P, Vanmanshoven P, Brouwers B, Vromans H, Georges M (1996). Identity-by-descent mapping of recessive traits in livestock: application to map the bovine syndactyly locus to chromosome 15. Genome Res.

[CR11] Citek J, Rehout V, Vecerek L, Hajkova J (2007). Genotyping glycogen storage disease type II and type V in cattle reared in the Czech Republic. J Vet Med A Physiol Pathol Clin Med.

[CR12] Dennis JA, Moran C, Healy PJ (2000). The bovine alpha-glucosidase gene: coding region, genomic structure, and mutations that cause bovine generalized glycogenosis. Mamm Genome.

[CR13] Drogemuller C, Leeb T, Harlizius B, Tammen I, Distl O, Holtershinken M, Gentile A, Duchesne A, Eggen A (2007). Congenital syndactyly in cattle: four novel mutations in the low density lipoprotein receptor-related protein 4 gene (LRP4). BMC Genet.

[CR14] Drogemuller C, Rossi M, Gentile A, Testoni S, Jorg H, Stranzinger G, Drogemuller M, Glowatzki-Mullis ML, Leeb T (2009). Arachnomelia in Brown Swiss cattle maps to chromosome 5. Mamm Genome.

[CR15] Drogemuller C, Tetens J, Sigurdsson S, Gentile A, Testoni S, Lindblad-Toh K, Leeb T (2010). Identification of the bovine Arachnomelia mutation by massively parallel sequencing implicates sulfite oxidase (SUOX) in bone development. PLoS Genet.

[CR16] Duchesne A, Gautier M, Chadi S, Grohs C, Floriot S, Gallard Y, Caste G, Ducos A, Eggen A (2006). Identification of a doublet missense substitution in the bovine LRP4 gene as a candidate causal mutation for syndactyly in Holstein cattle. Genomics.

[CR17] East L, McCarthy A, Wienke D, Sturge J, Ashworth A, Isacke CM (2003). A targeted deletion in the endocytic receptor gene Endo180 results in a defect in collagen uptake. EMBO Rep.

[CR18] Esmailizadeh AK, Bottema CD, Sellick GS, Verbyla AP, Morris CA, Cullen NG, Pitchford WS (2008). Effects of the myostatin F94L substitution on beef traits. J Anim Sci.

[CR19] Fasquelle C, Sartelet A, Li W, Dive M, Tamma N, Michaux C, Druet T, Huijbers IJ, Isacke CM, Coppieters W et al. (2009). Balancing selection of a frame-shift mutation in the MRC2 gene accounts for the outbreak of the Crooked Tail Syndrome in Belgian Blue Cattle. PLoS Genet 5 10.1371/journal.pgen.1000666 PMC2739430 19779552

[CR20] Gengler N, Seutin C, Boonen F, Van Vleck LD (1995). Estimation of genetic parameters for growth, feed consumption, and conformation traits for double-muscled Belgian blue bulls performance-tested in Belgium. J Anim Sci.

[CR21] Gill JL, Bishop SC, McCorquodale C, Williams JL, Wiener P (2009). Associations between the 11-bp deletion in the myostatin gene and carcass quality in Angus-sired cattle. Anim Genet.

[CR22] Greenberg CC, Jurczak MJ, Danos AM, Brady MJ (2006). Glycogen branches out: new perspectives on the role of glycogen metabolism in the integration of metabolic pathways. Am J Physiol Endocrinol Metab.

[CR23] Grisolia AB, D’Angelo GT, Porto Neto LR, Siqueira F, Garcia JF (2009). Myostatin (GDF8) single nucleotide polymorphisms in Nellore cattle. Genet Mol Res.

[CR24] Grobet L, Martin LJR, Poncelet D, Pirottin D, Brouwers B, Riquet J, Schoeberlein A, Dunner S, Menissier F, Massabanda J (1997). A deletion in the bovine myostatin gene causes the double-muscled phenotype in cattle. Nat Genet.

[CR25] Handschin C, Chin S, Li P, Liu F, Maratos-Flier E, Lebrasseur NK, Yan Z, Spiegelman BM (2007). Skeletal muscle fiber-type switching, exercise intolerance, and myopathy in PGC-1alpha muscle-specific knock-out animals. J Biol Chem.

[CR26] Jenkins MM, LeBoeuf RD, Ruth GR, Bloomer JR (1998). A novel stop codon mutation (X417L) of the ferrochelatase gene in bovine protoporphyria, a natural animal model of the human disease. Biochim Biophys Acta.

[CR27] Jiao S, Chu Q, Wang Y, Xie Z, Hou S, Liu A, Wu H, Liu L, Geng F, Wang C (2013). Identification of the causative gene for Simmental arachnomelia syndrome using a network-based disease gene prioritization approach. PLoS One.

[CR28] Johnson EB, Steffen DJ, Lynch KW, Herz J (2006). Defective splicing of Megf7/Lrp4, a regulator of distal limb development, in autosomal recessive mulefoot disease. Genomics.

[CR29] Josephsen K, Praetorius J, Frische S, Gawenis LR, Kwon TH, Agre P, Nielsen S, Fejerskov O (2009). Targeted disruption of the Cl/HCO^3^ exchanger Ae2 results in osteopetrosis in mice. Proc Natl Acad Sci USA.

[CR30] Kambadur R, Sharma M, Smith TP, Bass JJ (1997). Mutations in myostatin (GDF8) in double-muscled Belgian Blue and Piedmontese cattle. Genome Res.

[CR31] Karisa BK, Thomson J, Wang Z, Stothard P, Moore SS, Plastow GS (2013). Candidate genes and single nucleotide polymorphisms associated with variation in residual feed intake in beef cattle. J Anim Sci.

[CR32] Koltes JE, Mishra BP, Kumar D, Kataria RS, Totir LR, Fernando RL, Cobbold R, Steffen D, Coppieters W, Georges M (2009). A nonsense mutation in cGMP-dependent type II protein kinase (PRKG2) causes dwarfism in American Angus cattle. Proc Natl Acad Sci.

[CR33] Latter M, Latter B, Wilkins J, Windsor P (2006). Inheritance of proportionate dwarfism in Angus cattle. Aust Vet J.

[CR34] Lee SJ, McPherron AC (2001). Regulation of myostatin activity and muscle growth. Proc Natl Acad Sci USA.

[CR35] Leipold HW, Schmidt GL, Steffen DJ, Vestweber JG, Huston K (1998). Hereditary syndactyly in Angus cattle. J Vet Diagn Invest.

[CR36] Leisle L, Ludwig CF, Wagner FA, Jentsch TJ, Stauber T (2011). CIC-7 is a slowly voltage-gated 2CI(-)/1H(+)-exchanger and requires Ostm1 for transport activity. Embo J.

[CR37] Marchitelli C, Savarese MC, Crisa A, Nardone A, Marsan PA, Valentini A (2003). Double muscling in Marchigiana beef breed is caused by a stop codon in the third exon of myostatin gene. Mamm Genome.

[CR38] McPherron AC, Lawler AM, Lee SJ (1997). Regulation of skeletal muscle mass in mice by a new TGF-beta superfamily member. Nature.

[CR39] Meyers SN, McDaneld TG, Swist SL, Marron BM, Steffen DJ, O’Toole D, O’Connell JR, Beever JE, Sonstegard TS, Smith TP (2010). A deletion mutation in bovine SLC4A2 is associated with osteopetrosis in Red Angus cattle. BMC Genomics.

[CR40] Moreland RJ, Higgins S, Zhou A, VanStraten P, Cauthron RD, Brem M, McLarty BJ, Kudo M, Canfield WM (2012). Species-specific differences in the processing of acid alpha-glucosidase are due to the amino acid identity at position 201. Gene.

[CR41] Murgiano L, Jagannathan V, Benazzi C, Bolcato M, Brunetti B, Muscatello LV, Dittmer K, Piffer C, Gentile A, Drögemüller C (2014). Deletion in the EVC2 gene causes chondrodysplastic dwarfism in Tyrolean Grey cattle. PloS One.

[CR42] Muscatello L, Benazzi C, Dittmer K, Thompson K, Murgiano L, Drögemüller C, Avallone G, Gentile A, Edwards J, Piffer C (2015). Ellis–van Creveld Syndrome in Grey Alpine Cattle Morphologic, Immunophenotypic, and Molecular Characterization. Vet Pathol.

[CR43] Nicholas FW (2003). Online Mendelian Inheritance in Animals (OMIA): a comparative knowledgebase of genetic disorders and other familial traits in non-laboratory animals. Nucleic Acids Res.

[CR44] Nietfield J (2007) Osteopetrosis in calves. Diagnostic Insights 3

[CR45] O’Rourke BA, Dennis JA, Healy PJ (2006). Internal restriction sites: quality assurance aids in genotyping. J Vet Diagn Invest.

[CR46] O’Rourke BA, Greenwood PL, Arthur PF, Goddard ME (2013). Inferring the recent ancestry of myostatin alleles affecting muscle mass in cattle. Anim Genet.

[CR47] O’Toole D, Swist S, Steadman L, Johnson G (2012). Neuropathology and craniofacial lesions of osteopetrotic Red Angus calves. Vet Pathol.

[CR48] Olivan M, Martinez A, Osoro K, Sanudo C, Panea B, Olleta JL, Campo MM, Oliver MA, Serra X, Gil M (2004). Effect of muscular hypertrophy on physico-chemical, biochemical and texture traits of meat from yearling bulls. Meat Sci.

[CR49] Sanudo C, Macie ES, Olleta JL, Villarroel M, Panea B, Alberti P (2004). The effects of slaughter weight, breed type and ageing time on beef meat quality using two different texture devices. Meat Sci.

[CR50] Sartelet A, Druet T, Michaux C, Fasquelle C, Géron S, Tamma N, Zhang Z, Coppieters W, Georges M, Charlier C (2012). A splice site variant in the bovine RNF11 gene compromises growth and regulation of the inflammatory response. PLoS Genet.

[CR51] Sartelet A, Klingbeil P, Franklin CK, Fasquelle C, Geron S, Isacke CM, Georges M, Charlier C (2012). Allelic heterogeneity of Crooked Tail Syndrome: result of balancing selection?. Anim Genet.

[CR52] Sartelet A, Stauber T, Coppieters W, Ludwig CF, Fasquelle C, Druet T, Zhang Z, Ahariz N, Cambisano N, Jentsch TJ (2014). A missense mutation accelerating the gating of the lysosomal Cl/H^+^-exchanger ClC-7/Ostm1 causes osteopetrosis with gingival hamartomas in cattle. Dis Model Mech.

[CR53] Segovia-Silvestre T, Neutzsky-Wulff AV, Sorensen MG, Christiansen C, Bollerslev J, Karsdal MA, Henriksen K (2009). Advances in osteoclast biology resulting from the study of osteopetrotic mutations. Hum Genet.

[CR54] Seichter D, Russ I, Forster M, Medugorac I (2011). SNP-based association mapping of Arachnomelia in Fleckvieh cattle. Anim Genet.

[CR55] Sellick GS, Pitchford WS, Morris CA, Cullen NG, Crawford AM, Raadsma HW, Bottema CD (2007). Effect of myostatin F94L on carcass yield in cattle. Anim Genet.

[CR56] Sevane N, Nute G, Sanudo C, Cortes O, Canon J, Williams JL, Dunner S, Consortium G (2014). Muscle lipid composition in bulls from 15 European breeds. Livest Sci.

[CR57] Shi Z, Ferreira GC (2003). A continuous anaerobic fluorimetric assay for ferrochelatase by monitoring porphyrin disappearance. Anal Biochem.

[CR58] Shibuya H, Nonneman D, Tamassia M, Allphin OL, Johnson GS (1995). The coding sequence of the bovine ferrochelatase gene. Biochim Biophys Acta.

[CR59] Short RE, MacNeil MD, Grosz MD, Gerrard DE, Grings EE (2002). Pleiotropic effects in Hereford, Limousin, and Piedmontese F2 crossbred calves of genes controlling muscularity including the Piedmontese myostatin allele. J Anim Sci.

[CR60] Simon-Chazottes D, Tutois S, Kuehn M, Evans M, Bourgade F, Cook S, Davisson MT, Guenet JL (2006). Mutations in the gene encoding the low-density lipoprotein receptor LRP4 cause abnormal limb development in the mouse. Genomics.

[CR61] Smith TP, Lopez-Corrales NL, Kappes SM, Sonstegard TS (1997). Myostatin maps to the interval containing the bovine mh locus. Mamm Genome.

[CR62] Soethout EC, Verkaar EL, Jansen GH, Muller KE, Lenstra JA (2002). A direct StyI polymerase chain reaction-restriction fragment length polymorphism (PCR-RFLP) test for the myophosphorylase mutation in cattle. J Vet Med A Physiol Pathol Clin Med.

[CR63] Stark Z, Savarirayan R (2009). Osteopetrosis. Orphanet J Rare Dis.

[CR64] Straka JG, Hill HD, Krikava JM, Kools AM, Bloomer JR (1991). Immunochemical studies of ferrochelatase protein: characterization of the normal and mutant protein in bovine and human protoporphyria. Am J Hum Genet.

[CR65] Teitelbaum SL (2000). Bone resorption by osteoclasts. Science.

[CR66] Testoni S, Gentile A (2004). Arachnomelia in four Italian brown calves. Vet Rec.

[CR67] Tolar J, Teitelbaum SL, Orchard PJ (2004). Osteopetrosis. N Engl J Med.

[CR68] Whitlock BK, Kaiser L, Maxwell HS (2008). Heritable bovine fetal abnormalities. Theriogenology.

[CR69] Yadegari M, Vahed E, Ashtari M, Tavakol S, Khamesipour F (2013) Report of congenital syndactyly (mule foot) in cattle

[CR70] Zlotowski P, Gimeno EJ, Diaz A, Barros R, Barros SS, Cruz CE, Driemeier D (2006). Lectin-histochemistry: glycogenosis in cattle. Vet Res Commun.

